# Risk factors for refractory enterocutaneous fistula following button jejunostomy removal and its treatment using a novel extraperitoneal approach in patients with oesophageal cancer: a retrospective cohort study

**DOI:** 10.1186/s12876-022-02524-2

**Published:** 2022-11-25

**Authors:** Teppei Kamada, Hironori Ohdaira, Keigo Nakashima, Ryo Nishide, Junji Takahashi, Eisaku Ito, Yuichi Nakaseko, Norihiko Suzuki, Masashi Yoshida, Ken Eto, Yutaka Suzuki

**Affiliations:** 1 Department of Surgery, International University of Health and Welfare Hospital, 537-3, 329-2763 Iguchi, Nasushiobara, Tochigi Japan; 2 Department of Surgery, The Jikei University School of Medicine, 3-25-8, Nishi-shimbashi, Minato-ku, 105-8461 Tokyo, Japan

**Keywords:** Button jejunostomy, Refractory enterocutaneous fistula, Fistula closure, Oesophagectomy

## Abstract

**Background:**

Enterocutaneous fistula after removal of the jejunostomy tube leads to multiple problems, such as cosmetic problems, decreased quality of life, electrolyte imbalances, infectious complications, and increased medical costs. However, the risk factors for refractory enterocutaneous fistula (REF) after button jejunostomy removal remain unclear. Therefore, in this study, we assessed the risk factors for REF after button jejunostomy removal in patients with oesophageal cancer and reported the surgical outcomes of the novel extraperitoneal approach (EPA) for REF closure.

**Methods:**

This retrospective cohort study included 47 patients who underwent button jejunostomy removal after oesophagectomy for oesophageal cancer. We assessed the risk factors for REF in these patients and reported the surgical outcomes of the novel EPA for REF closure at the International University of Health and Welfare Hospital between March 2013 and October 2021. The primary endpoint was defined as the occurrence of REF after removal of the button jejunostomy, which was assessed using a maintained database. The risk factors and outcomes of the EPA for REF closure were retrospectively analysed.

**Results:**

REFs occurred in 15 (31.9%) patients. In the univariate analysis, REF was significantly more common in patients with albumin level < 4.0 g/dL (p = 0.026), duration > 12 months for button jejunostomy removal (p = 0.003), and with a fistula < 15.0 mm (p = 0.002). The multivariate analysis revealed that a duration > 12 months for button jejunostomy removal (odds ratio [OR]: 7.15; 95% confidence interval [CI]: 1.38–36.8; p = 0.019) and fistula < 15.0 mm (OR: 8.08; 95% CI: 1.50–43.6; p = 0.002) were independent risk factors for REF. EPA for REF closure was performed in 15 patients. The technical success rate of EPA was 88.2%. Of the 15 EPA procedures, fistula closure was achieved in 12 (80.0%). The complications of EPA (11.7%) were major leakages (n = 3) and for two of them, EPA procedure was re-performed, and closure of the fistula was finally achieved.

**Conclusion:**

This study suggested that duration > 12 months for button jejunostomy removal and fistula < 15.0 mm are the independent risk factors for REF after button jejunostomy removal. EPA for REF closure is a novel, simple, and useful surgical option for patients with REF after oesophagectomy.

## Background

Early enteral nutrition is recommended for peri-operative management of oesophageal cancers to reduce complications [1]. Feeding jejunostomy is a common pathway for enteral nutrition after oesophagectomy, and it is not uncommon to require enteral nutrition by feeding jejunostomy for a long time, not only in the peri-operative period but also for anorexia and weight loss due to recurrences and chemotherapy adverse events [2, 3]. However, serious complications of feeding jejunostomy, such as bowel obstruction, abdominal wall infection, tube deviation, and peritonitis owing to fistula injury during replacement, have been reported [4–6]. In particular, enterocutaneous fistula after removal of the jejunostomy tube leads to multiple problems, such as cosmetic problems, decreased quality of life, electrolyte imbalances, infectious complications, and increased medical costs.

Button jejunostomy has cosmetic advantages and employs an easily replaceable feeding button compared with the conventional Witzel jejunostomy [7, 8]. Conversely, it has been reported that refractory enterocutaneous fistulas (REFs) are more common after removal of button jejunostomy than Witzel jejunostomy [9]. Few studies have reported the risk factors of REFs after button jejunostomy removal. In addition, there are few reports of effective minimally invasive treatments for REFs that failed to close the defect spontaneously.

We introduced button jejunostomy in oesophagectomy for oesophageal cancer in 2010, and have been performing a novel extraperitoneal approach for closure of REFs. In this study, we examined the risk factors for REFs after removal of button jejunostomy in patients with oesophageal cancer and reported the surgical outcomes of the extraperitoneal approach (EPA) for closure of REFs.

## Methods

### Patient selection

**Fig. 1:**
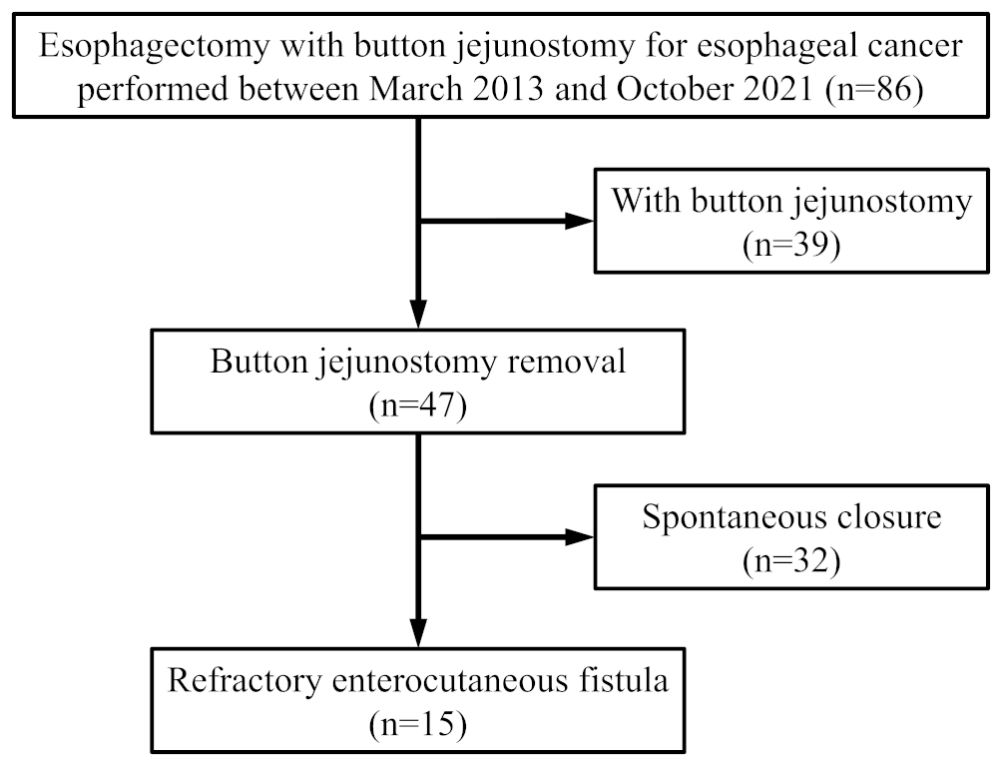
Patient selection for the study Of the 86 patients who underwent radical oesophagectomy with button jejunostomy, button jejunostomy removal was performed in 47 patients and refractory enterocutaneous fistula occurred in 15 patients

This retrospective cohort study included 47 patients who underwent button jejunostomy removal after oesophagectomy for oesophageal cancer at the International University of Health and Welfare Hospital (Nasushiobara, Tochigi Prefecture, Japan) between March 2013 and October 2021 (Fig. 1). We aimed to assess the risk factors for REFs in them and to evaluate the surgical outcomes of EPA for closure of REFs.We retrospectively examined the primary endpoint, which was defined as the occurrence of REF after button jejunostomy removal, using a maintained database. We included patients in whom button jejunostomy was created during oesophagectomy for oesophageal cancer and removed after surgery with complete follow-up data and clinical details available. Patients who underwent direct percutaneous endoscopic jejunostomy and those who underwent Witzel jejunostomy were excluded.

This study was conducted in accordance with the Declaration of Helsinki and approved by the Institutional Review Board of the International University of Health and Welfare Hospital (IR number: 22-B-16). Informed consent was taken from all patients in the form of giving opportunity to opt out their records from being used in the analyses.

### Jejunostomy placement and post-operative feeding (Fig. 2a)

**Fig. 2:**
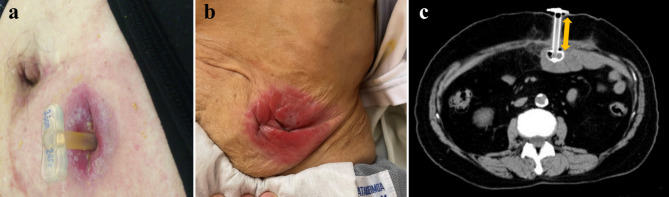
(a) Button jejunostomy (b) Skin erosion in refractory enterocutaneous fistula after button jejunostomy removal (c) Computer tomography image showing measurement of the fistula length. The fistula length is measured as the vertical distance between the skin and intestinal puncture site

Button jejunostomy was performed in the same method in all patients during oesophagectomy. The details of button jejunostomy creation were described in a previous report [6]. We inserted a 24-Fr button-type catheter (IDEAL BUTTON®; Olympus Medical Systems Co.Ltd, Tokyo, Japan) to the jejunum. The appropriate length of the button-type catheter was determined based on the thickness of the abdominal wall. Continuous enteral nutrition was initiated from the first post-operative day at a rate of 10 mL/h and gradually increased to 60–80 mL/h, according to the gradual decrease in the intravenous fluid administration. After discharge, supportive enteral nutrition at 400–800 kcal/day was continued, according to the oral intake. Replacement of the jejunostomy button was performed every 4–6 months. When it was judged that supportive enteral nutrition was not required using blood investigations, imaging, and body weight evaluation, removal of the jejunostomy button was performed.

### Definition of REF (Fig. 2b)

REF was defined as a condition, in which spontaneous closure of the fistula was not achieved after removal of the button jejunostomy with continuous leakage and requirement of surgical treatment.

### Measurement of the Fistula length (Fig. 2c)

The length of the fistula was measured as the vertical distance between the skin and intestinal puncture site in the coronal section using computed tomography performed within 1 month before button jejunostomy removal.

### EPA for REF closure (Fig. 3)

**Fig. 3:**
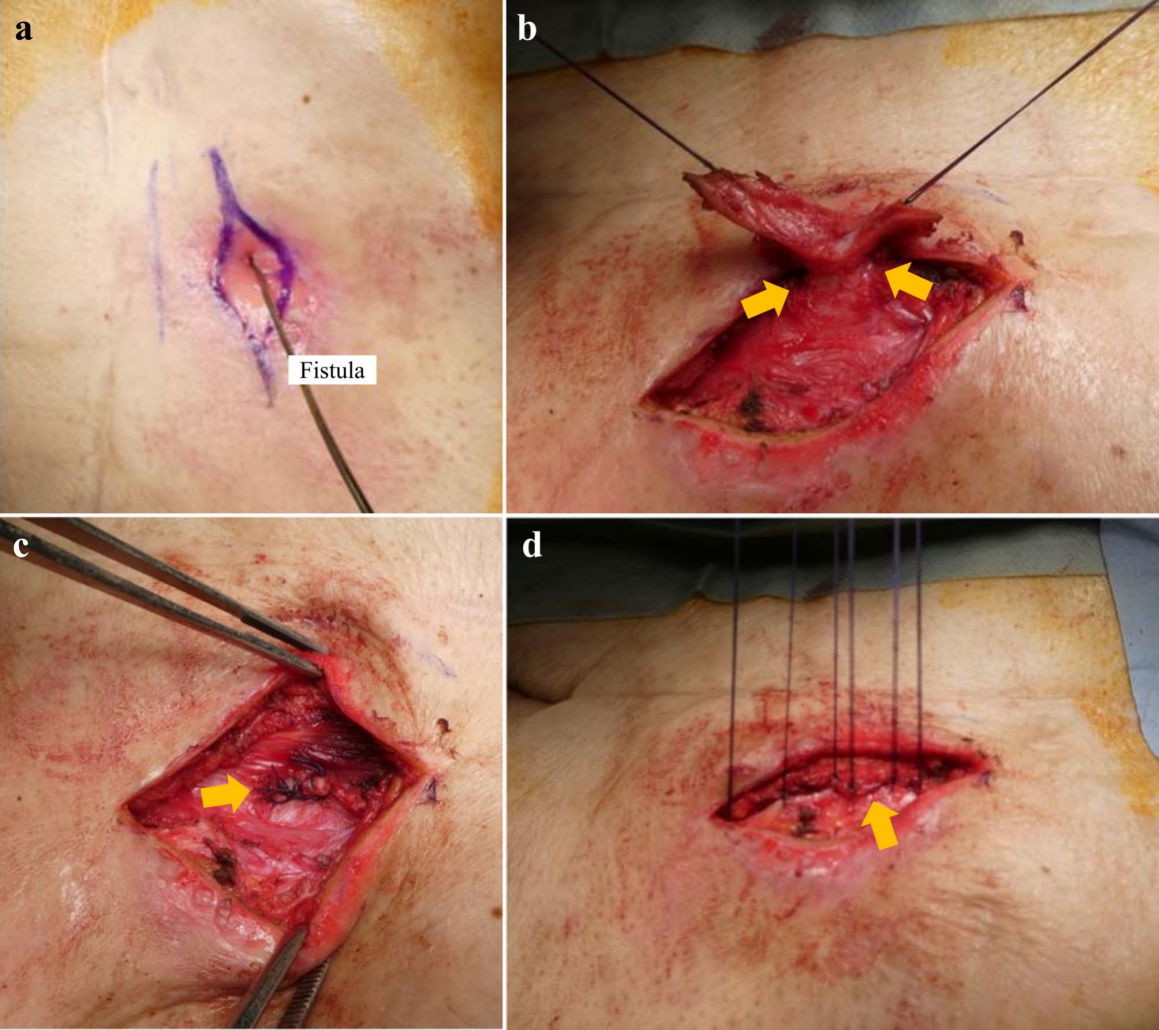
Extraperitoneal approach for REF closure. (a) Identification of the fistula using a probe (b) Separation of the fistula from the surrounding tissue (c) Transection of the fistula by double ligation using absorbable sutures (d) Closing the anterior layer of rectus sheath

The EPA for REF closure was performed by two surgeons, with the patient being in the supine position. Local, general, or spinal anaesthesia was administered according to the patients’ surgical tolerance.

1. The fistula was identified using a probe, and a spindle-shaped incision of approximately 3–5 cm around the fistula was created (Fig. 3a).
2. The fistula was separated from the surrounding tissue as deeply as possible, paying attention to the injury to the fistula and its arrival into the abdominal cavity (Fig. 3b).
3. The fistula was transected by double ligation using absorbable sutures (Fig. 3c).
4. The rectus abdominis muscle was released followed by covering it on the transected fistula.
5. The anterior layer of the rectus sheath and subcutaneous tissue were closed using absorbable sutures and the epidermis was closed using a skin stapler (Fig. 3d).

### Statistical analysis

The Mann–Whitney U test, unpaired t-test, and chi-squared test were used to compare the continuous and dichotomous categorical variables. Multivariate analyses were performed using a multivariate logistic regression model. The cut-off levels of each variable in the univariate and multivariate analyses were defined as the values that maximised the Youden index for predicting the REF on each receiver operating characteristic curve. Statistical significance was set at p < 0.05. STATA/IC version 16.0 (STATA Statistical Software; StataCorp, College Station, TX, USA) was used for statistical analyses.

## Results

In total, 47 patients were enrolled in this study. The patients’ baseline characteristics and oncological and peri-operative factors are summarised in Table 1. The study included 41 men and six women, with a median age of 70.4 ± 9.4 years. The median body mass index was 19.9 ± 3.1, and the histological types were squamous cell carcinoma in 41 (87.2%) patients and others in six (12.8%) patients. Thoracic, abdominal, and cervical oesophageal cancers were present in 36 (76.6%), nine (19.2%), and two (4.3%) patients, respectively. The pathological stages were as follows: stage I, n = 26 (55.3%); stage II, n = 13 (27.7%); and stage III, n = 8 (17.0%). Post-operative adjuvant chemotherapy was administered to 28 (59.6%) patients.

**Table 1:** Demographic and clinicopathological characteristics of patient cohort

Variable		Total
Patients, number		47
Age, years		70.4 ± 9.4
Sex	Men	41 (87.2%)
	Women	6 (12.8%)
Body mass index, kg/m^2^		19.9 ± 3.1
Histopathology, n (%)		
Squamous cell carcinoma		41 (87.2%)
Others		6 (12.8%)
Primary tumour location, n (%)		
Cervical		2 (4.3%)
Thoracic		36 (76.6%)
Abdominal		9 (19.2%)
Pathological stage, n (%)		
I		26 (55.3%)
II		13 (27.7%)
III		8 (17.0%)
Pre-operative chemotherapy		7 (14.9%)
Pre-operative radiotherapy		2 (4.3%)
Adjuvant chemotherapy		28 (59.6%)
Operation time, min		456.7 ± 62.9
Intra-operative blood loss, mL		158.2 ± 192.3
Diabetes mellitus		6 (12.8%)
Current smoking		25 (53.2%)
Sarcopenia		19 (40.4%)
HALS		17 (36.2%)
Complete laparoscopic surgery		27 (57.4%)
Robot assisted surgery		3 (6.4%)
Pneumonia		12 (25.3%)
Anastomotic leakage		7 (14.8%)
Ileus		6 (12.8%)
RLNP		12 (25.5%)
Refractory enterocutaneous fistula		15 (31.9%)
Recurrence		9 (19.2%)
Death		11 (23.4%)
Length of fistula, mm		16.3 ± 6.4
Duration for removal jejunostomy, months		12.6 ± 7.0

Data are expressed as means ± standard deviations and numbers (%)

HALS, hand-assisted laparoscopic surgery; RLNP, recurrent laryngeal nerve paralysis

Post-operative pneumonia was observed in 12 (25.3%), anastomotic leakage in seven (14.8%), recurrent laryngeal nerve paralysis in 12 (25.5%), ileus in six (12.8%), and REF in 15 (31.9%) patients. The median length of the fistula was 16.3 ± 6.4 mm, and the median duration for button jejunostomy removal was 12.6 ± 7.0 months. The mean follow-up period was 46.4 (2.4–91.2) months. During the follow-up period, nine (19.2%) patients relapsed and 11 (23.4%) patients died.

### Risk factors for REF

The 47 patients were divided into the REF and non-REF groups (Table 2). In the univariate analysis, REF was significantly more common in patients with albumin level < 4.0 g/dL (p = 0.026), duration for button jejunostomy removal > 12 months (p = 0.003), and fistula < 15.0 mm (p = 0.002). All variables that demonstrated a significant difference in the univariate analysis were included in the multivariate analysis using a logistic regression model. In the multivariate analysis, duration for button jejunostomy removal > 12 months (odds ratio [OR]: 7.15; 95% confidence interval [CI]:1.38–36.8; p = 0.019) and fistula length < 15.0 mm (OR: 8.08; 95% CI: 1.50–43.6; p = 0.002) were found to be independent risk factors for REF.

**Table 2:** Pre- and peri-operative risk factors for refractory enterocutaneous fistula after button jejunostomy removal

Variable		REF	Non-REF	Univariate p-value	Multivariate OR (95% CI) p-value
		n (%) or median (range)			
Patients		15	32		
Age > 61, years		15 (100%)	27 (84.4%)	0.11	
Sex	Men	15 (100%)	26 (81.3%)	0.07	
	Women	0 (0%)	6 (18.8%)		
Body mass index < 9.0, kg/m^2^		8 (53.3%)	12 (37.5%)	0.31	
Primary tumour location				0.17	
Cervical		0 (0%)	2 (6.25%)		
Thoracic		14 (93.3%)	22 (68.75%)		
Abdominal		1 (6.7%)	8 (25.0%)		
Pathological stage				0.72	
I		7 (46.7%)	19 (59.4%)		
II		5 (33.3%)	8 (25.0%)		
III		3 (20.0%)	5 (15.6%)		
Adjuvant chemotherapy		10 (66.7%)	18 (56.3%)	0.49	
Operation time > 517 min		4 (26.7%)	3 (9.4%)	0.12	
Blood loss > 170 mL		6 (40.0%)	7 (21.9%)	0.19	
Diabetes mellitus		4 (26.7%)	2 (6.3%)	0.051	
Current smoking		9 (60.0%)	16 (50.0%)	0.52	
Anastomotic leakage		3 (20.0%)	4 (12.5%)	0.50	
Ileus		1 (6.7%)	5 (15.6%)	0.39	
Sarcopenia		8 (53.3%)	11 (34.4%)	0.22	
Recurrence		5 (33.3%)	4 (12.5%)	0.09	
Albumin level < 4.0 g/dL		13 (86.7%)	17 (53.1%)	0.026	6.31 (0.89–44.6) 0.065
Duration for removal > 12 months		11 (73.3%)	9 (28.1%)	0.003	7.15 (1.38–36.8) 0.019
Length of fistula < 15.0 mm		12 (80.0%)	10 (31.3%)	0.002	8.08 (1.50–43.6) 0.015

REF, refractory enterocutaneous fistula; CI, confidence interval; OR, odds ratio

### Outcomes of EPA for REF closure

The baseline characteristics and peri-operative clinical outcomes of patients with REF who underwent REF closure with EPA are presented in Table 3. The mean age was 72.2 years (61–91 years), and all were men. The mean length of the fistula was 12.7 (8.8–17.3) mm, and the mean duration for jejunostomy removal was 18.2 (2–29) months. The mean albumin level was 3.6 (3.0–4.2) g/dL. EPA was performed in 15 patients (17 procedures), and the conversion to bowel resection due to unexpected laparotomy during EPA was performed in two (11.7%) patients.

**Table 3:** Details of the 15 patients with refractory enterocutaneous fistula after button jejunostomy removal

Patients	Age (years)/ Sex	Size of the button (cm)	Length of fistula (mm)	Duration for removal (month)	Albumin level (g/dL)	Duration from removal to EPA (day)	Procedure	Operative time (min)	Anaesthesia	Complications	Hospital stay (day)
#1	61/M	24 Fr 4.0	18.0	20	3.7	24	EPA	14	Epidural	None	7
#2	64/M	24 Fr 4.0	12.8	17	4.2	39	EPA	26	Local	None	1
#3	91/M	24 Fr 3.0	10.0	11	3.3	27	EPA EPA	27 24	Local Local	Major leakage None	26 9
#4	69/M	24 Fr 3.5	12.7	14	4.1	30	EPA	19	Local	None	1
#5	68/M	24 Fr 4.0	13.9	26	3.3	51	EPA EPA	34 16	General General	Major leakage None	2 5
#6	72/M	24 Fr 4.0	11.4	25	3.6	57	EPA	52	General	None	12
#7	75/M	24 Fr 3.5	17.3	7	3.5	22	EPA	19	General + Epidural	None	5
#8	62/M	24 Fr 3.5	17.1	20	3.9	36	EPA	35	Local	None	1
#9	81/M	24 Fr 4.0	9.23	12	3.0	13	EPA	56	General	Major leakage	42
#10	72/M	24 Fr 3.0	9.93	15	3.0	9	EPA→ Bowel resection	88	General+ Epidural	Major leakage	27
#11	75/M	24 Fr 4.0	11.7	25	3.8	179	EPA	26	General	None	1
#12	63/M	24 Fr 4.5	8.8	23	3.9	62	EPA	22	General	None	2
#13	81/M	24 Fr 4.0	13.0	3	3.5	177	EPA	24	General	None	1
#14	78/M	24 Fr 3.5	13.9	29	3.9	93	EPA	50	General	None	7
#15	71/M	24 Fr 3.0	11.0	26	3.3	70	EPA→ Bowel resection	114	General	None	13

EPA, extraperitoneal approach

The technical success rate of EPA was 88.2% (15/17 cases). Two patients required bowel resection owing to a short fistula length (< 11.0 mm) and injury to the fistula. Local, epidural, and general anaesthesia was administered in five (29.4%), three (17.6%), and 10 (66.7%) patients (with duplication), respectively. The fistula closure was achieved in 12 (80.0%) of 15 EPAs. The mean operation time was 38 (14–114) min, and the mean length of hospital stay was 9.5 (1–42) days. The complication of EPA was major leakages (n = 3, 13.3%), and for two of them, EPA was re-performed, and fistula closure was finally achieved. All four patients, including one patient who had undergone bowel resection, with major leakage had (1) American Society of Anaesthesiologists score ≥ 3 points with coronary artery disease or chronic obstructive pulmonary disease, (2) cancer recurrence, (3) serum albumin level < 3.3 g/dL at the time of button jejunostomy removal, and (4) heavy smoking history.

## Discussion

Button jejunostomy is a novel jejunostomy creation technique first reported in 1989 [7–10]. There are some advantages of the button jejunostomy, including the simplicity of the procedure, ease of replacement, low risk of tube deviations and obstructions, patient’s aesthetic outcomes, and a high quality of life. However, a common complication is REF after button jejunostomy removal. No studies have shown the risk factors for RFF after button jejunostomy removal and the outcomes of the EPA for REF closure, until now.

In this study, the duration for button jejunostomy removal (> 12 months) and fistula length (< 15.0 mm) were found to be the independent risk factors for REF, and minimally invasive REF closure by the EPA had good outcomes.

There is no consensus on the timing of jejunostomy removal because the amount of oral intake varies depending on the patient’s primary disease stage and the presence of post-operative complications. However, according to a previous report [9], the risk of REF increased when the duration of jejunostomy removal was > 1 year, and the results of this study also supported the previous report. Epithelialisation of the fistula was considered to gradually form > 1 year, resulting in REF [11].

Since the fistula length was measured as the vertical distance between the skin and the intestinal puncture site, it was defined as the sum of the subcutaneous fat thickness plus the rectus abdominis muscle. However, the body mass index or presence of sarcopenia, which is an index of skeletal muscle mass, does not show a significant difference in the risk of REF; therefore, the fistula length should be considered as an independent risk factor. It has been reported that enterocutaneous fistulas, which close spontaneously, have long fistula tracts (> 2 cm) [12], and our results could be a useful cut-off value for REF in button jejunostomy with a short fistula length.

The length and thickness of the fistula are considered to be the reasons for frequent development of REF after button jejunostomy removal than after Witzel jejunostomy. The jejunostomy button used in our hospital is 24 Fr with a diameter of 8.0 mm, whereas the jejunal tube used in the Witzel jejunostomy is 10–12 Fr with a diameter of 4.0 mm. The diameter of the fistula was longer in button jejunostomy than in Witzel jejunostomy. Furthermore, in button jejunostomy, a fistula is formed vertically to the abdominal wall, but in Witzel jejunostomy, a diagonal fistula is formed; therefore, the length of the fistula in button jejunostomy is shorter than that in Witzel jejunostomy. It has been reported that enterocutaneous fistula that can be expected to spontaneously close have a diameter of ≤ 1 cm and length of ≥ 2 cm [12]. This report supports our hypothesis that REF occurs more frequently after button jejunostomy removal. In addition, ‘free distal flow’ has been reported as an important factor in spontaneous closure [11]. Button jejunostomy has an acute bending angle between the jejunostomy and abdominal wall compared with Wizel jejunostomy [6], which may interfere with the natural flow of intestinal fluid to the distal intestinal tract and lead to REF.

Spontaneous closure usually can be expected after removal of the jejunostomy tube; however spontaneous closure often requires 4–6 weeks [4, 11, 12]. Immediate fistula closure can be an effective option for patients waiting for spontaneous fistula closure that require a long period of time to close or those suffering from REF. There are some reports of minimally invasive treatments for REF, such as the use of over-the-scope clip [13] and fibrin glue [14]. The success rate is reported to be approximately 50–87.5%, but the applicable fistula is a small enterocutaneous fistula with a diameter of approximately 5 mm or a low-output enterocutaneous one. It is difficult to treat a high-output fistula with a large diameter, such as REF, after removing the jejunostomy button. Although bowel resection, including the fistula, is highly curative, it can be a highly invasive treatment for patients after oesophagectomy with severe intra-abdominal adhesions, malnutrition, and poor surgical tolerance.

For the aforementioned reasons, we devised an EPA to treat the REF. This procedure is simple, has a short operative time, can be performed even under local anaesthesia, does not reach the abdominal cavity, and has the advantage of not being affected by adhesions from the previous surgery. The fistula was transected by separating and ligating it as deep as possible on the intestinal side, reducing the pressure on the intestinal side of the fistula, and closing it by causing scarring at the transected point. In this procedure, we consider that the fistula can be easily separated from the surrounding tissue as deeply as possible with good visual field, and aesthetic outcomes of the wound can be improved along the skin dividing line by creating a spindle-shaped incision of approximately 3–5 cm around the fistula.

The success rate of EPA for REF was 80%, with good results. If the patient was judged to be operable under general anaesthesia, EPA was performed under general anaesthesia in case of unexpected fistula injury or laparotomy.

This study had some limitations. First, it was retrospective and was conducted at a single centre with a small number of patients. Further studies using data from a large-scale, multicentre registry should be conducted in the future. Second, this study is limited to button jejunostomy and may not be applicable to REF in other jejunostomy creation methods. Third, fistula closure using a single EPA may not be effective in high-risk patients with cancer recurrence or poor nutrition. In addition, the definition of REF remains controversial, and in this study, the timing of performing the EPA was not unified. Finally, considering that the high incidence rate of REF after removal of the button jejunostomy (31.9%), instead of performing button jejunostomy routinely, early removal of the button jejunostomy or Witzel jejunostomy should be considered depending on the patients’ risk.

## Conclusion

This study suggested that duration > 12 months for button jejunostomy removal and fistula < 15.0 mm are the independent risk factors for REF after button jejunostomy removal. The technique used in this study, EPA for REF closure, is a novel, simple, and useful surgical option for patients with REF after oesophagectomy.

## Data Availability

The datasets generated and/or analysed during the current study are not publicly available due to the anonymity of the participants but are available from the corresponding author on reasonable request.

## Abbreviations

REF: Refractory enterocutaneous fistula
EPA: Extraperitoneal approach
OR: Odds ratio
CI: CIConfidence interval
